# Therapeutic Potential of TAAR1 Agonists in Schizophrenia: Evidence from Preclinical Models and Clinical Studies

**DOI:** 10.3390/ijms222413185

**Published:** 2021-12-07

**Authors:** Nina Dedic, Heather Dworak, Courtney Zeni, Grazia Rutigliano, Oliver D. Howes

**Affiliations:** 1 Sunovion Pharmaceuticals, Marlborough, MA 01752, USA; heather.dworak@sunovion.com (H.D.); courtney.zeni@sunovion.com (C.Z.); 2 Department of Pathology, University of Pisa, via Savi 10, 56126 Pisa, Italy; grazia.rutigliano@unipi.it; 3 Institute of Clinical Sciences, Faculty of Medicine, Imperial College London, London SW7 2AZ, UK; oliver.howes@kcl.ac.uk; 4 Department of Psychosis Studies, Institute of Psychiatry, Psychology & Neuroscience, Kings College London, London SE5 8AF, UK; 5 Psychiatric Imaging Group, Medical Research Council, London Institute of Medical Sciences, Hammersmith Hospital, London W12 0NN, UK

**Keywords:** trace amines, trace amine-associated receptor 1 (TAAR1), schizophrenia, psychosis, dopamine, serotonin, glutamate, neurocognition

## Abstract

Trace amine-associated receptor 1 (TAAR1) has emerged as a promising therapeutic target for neuropsychiatric disorders due to its ability to modulate monoaminergic and glutamatergic neurotransmission. In particular, agonist compounds have generated interest as potential treatments for schizophrenia and other psychoses due to TAAR1-mediated regulation of dopaminergic tone. Here, we review unmet needs in schizophrenia, the current state of knowledge in TAAR1 circuit biology and neuropharmacology, including preclinical behavioral, imaging, and cellular evidence in glutamatergic, dopaminergic and genetic models linked to the pathophysiology of psychotic, negative and cognitive symptoms. Clinical trial data for TAAR1 drug candidates are reviewed and contrasted with antipsychotics. The identification of endogenous TAAR1 ligands and subsequent development of small-molecule agonists has revealed antipsychotic-, anxiolytic-, and antidepressant-like properties, as well as pro-cognitive and REM-sleep suppressing effects of TAAR1 activation in rodents and non-human primates. Ulotaront, the first TAAR1 agonist to progress to randomized controlled clinical trials, has demonstrated efficacy in the treatment of schizophrenia, while another, ralmitaront, is currently being evaluated in clinical trials in schizophrenia. Coupled with the preclinical findings, this provides a rationale for further investigation and development of this new pharmacological class for the treatment of schizophrenia and other psychiatric disorders.

## 1. Introduction

### 1.1. Schizophrenia: A Severe Psychiatric Disease with Significant Unmet Needs

Schizophrenia is a severe, chronic, and often disabling psychiatric disorder affecting nearly 20 million people worldwide [1]. It is characterized by symptoms grouped into positive, negative, and cognitive domains with patients experiencing varying levels of each (Table 1). 

Schizophrenia remains one of the most challenging diseases to treat, as significant heterogeneity exists within the illness and with regard to treatment response. For most patients, the first episode of psychosis occurs in late adolescence or early adulthood, and is frequently preceded by a prodromal phase comprised of cognitive and social deficits or challenges [4]. In general, positive symptoms occur in a relapsing–remitting fashion, while negative and cognitive symptoms tend to be more chronic with an increasing impact on social functioning. 

Over half (56.0%) of individuals with schizophrenia have co-occurring mental and/or behavioral health disorders (including drug abuse/dependency (29.0%), depression (27.7%), alcohol abuse/dependency (24.6%), and anxiety disorder (13.6%)) leading to additional treatment challenges, increased rates of functional impairment, and rates of unemployment above 75% [5,6,7]. Schizophrenia is also associated with an increased risk of premature mortality (>10-year reduction in average life expectancy), primarily due to increased cardiovascular disease and increased suicide rates [8,9,10]. The diagnosis of schizophrenia is associated with an increase in cardiovascular risk factors (e.g., obesity, hypertension, diabetes, dyslipidemia), likely attributable not only to an unhealthy and sedentary lifestyle, but also to the adverse effects of some antipsychotic medication on weight and metabolic parameters [11,12]. 

Whether a single unifying pathophysiological process is shared across patients or different independent disease processes lead to a similar clinical syndrome remains a crucial focus of research [13]. Several etiological hypotheses of schizophrenia have been proposed based on evidence for the contribution of genetic risk factors [14], neurodevelopmental alterations [15], immune disturbances and inflammation [16,17,18,19], trauma [20], socioeconomic status and urbanization [21,22], and last but not least, dysfunction in several neurotransmitter and neuropeptide systems (reviewed by Kaar et al.) [23]. 

For many years, the prevailing neurotransmitter-based theory of schizophrenia has centered on the dopamine (DA) hypothesis, linking psychotic symptoms to hyperactivity of the DA mesolimbic system. The DA hypothesis arose in the 1960s after studies showed that antipsychotics block dopamine D_2_-receptors. This, together with the observation that drugs that potentiate dopamine neurotransmission, such as amphetamine, can mimic psychotic symptoms in healthy individuals [24], solidified the basis for much of the DA hypothesis. More recently, neuroimaging studies have shown increased presynaptic DA synthesis capacity in the striatum of patients with schizophrenia, which also correlates with psychotic symptom severity [25,26,27,28]. In addition to dopaminergic dysfunction, changes in serotonin (5-hydroxytryptamine [5-HT]), glutamate, and other neurotransmitters have also been associated with the pathophysiology of schizophrenia [29,30]. In particular, cortical N-Methyl-D-aspartic acid (NMDA) receptor hypofunction [31,32,33] as well as serotonin 5-HT_2A_ hyperfunction [34,35] has been linked to psychosis. Although initially investigated separately, the outlined hypotheses are becoming increasingly integrated, including evidence that glutamatergic dysregulation in cortical regions can lead to striatal dopamine dysfunction, and ultimately to the development of psychosis [36].

### 1.2. Current Pharmacologic Treatments 

Antipsychotics are the pharmacological standard of care for schizophrenia globally [37,38,39]. Despite the complexity of the disease, current drug treatments primarily rely on one mechanism of action: dopamine D_2_ receptor blockade. First-generation antipsychotics, discovered initially in the 1950s, exert their action predominantly through dopamine D_2_ receptor antagonism. While D_2_ receptor antagonism accounts for the therapeutic efficacy of these agents, it is also associated with adverse side effects including hyperprolactinemia and extrapyramidal symptoms (EPS) including tardive dyskinesia, acute dystonias, parkinsonism, and akathisia [37,38]. Second-generation antipsychotics, largely introduced into the clinic in the 1990s, often exert additional antagonism at other receptors such as 5-HT_2A_ [23]. This receptor profile coupled with dosing to avoid unnecessarily high D2 occupancy has resulted in lower overall risk for hyperprolactinemia and EPS [40], as demonstrated for agents such as clozapine, olanzapine, quetiapine, aripiprazole and lurasidone relative to first generation antipsychotics. Most antipsychotics exhibit a dose-dependent relationship between D_2_ receptor occupancy and their therapeutic effects [23]. Several antipsychotics also demonstrate additional activity at adrenergic, cholinergic, histaminergic and/or other serotonergic receptors. Although these additional targets may confer therapeutic effects, they may also contribute to aspects of their side effect profiles. For example, adrenergic (α1) receptor antagonism is associated with orthostatic hypotension, blockade of histamine (H_1_) receptors is linked to sedation and weight gain, and muscarinic (M_1_) receptor blockade is associated with dry mouth, blurred vision and constipation [23,38,41]. While variable among antipsychotics, metabolic dysregulation (including body weight gain) is highly prevalent [11,42,43]. The underlying cause remains largely elusive, with activity at several targets being implicated in antipsychotic-induced weight gain and disruption of glucose/insulin homeostasis, including serotonin 5-HT_2C_/5-HT_2A_, histamine H_1_, dopamine D_1_/D_2_/D_3_, adrenergic α1 and muscarinic M_3_ receptors [44,45,46]. 

Currently available antipsychotic agents are effective in treating the positive symptoms of schizophrenia (e.g., hallucinations, delusions, and disorganized thinking), however, they largely leave the negative (e.g., anhedonia, alogia, and avolition) and cognitive symptom domains (e.g., problems with verbal and visual learning, problem solving, and attention) untreated [47,48,49,50,51]. Although negative symptom prevalence varies across studies and definitions, up to 60% of patients have been categorized as having prominent or predominant negative symptoms that require treatment [48]. While several antipsychotics have demonstrated negative symptom improvement in patients with an acute exacerbation of schizophrenia, these clinical trials were designed to study patients defined by positive symptoms, and therefore treatment response cannot be easily teased apart from secondary negative symptom improvement as a result of improvement in other domains [48]. Strategies to combat this deficit in pharmacologic treatment, such as combining antipsychotics or adding other types of adjunctive agents, have largely been unsuccessful. 

Comparative effectiveness studies such as CATIE, CUTLASS and EUFEST have shown a large degree of similarity between older and newer antipsychotic agents, with side effects being the greatest discriminating factor [52,53,54]. The only exception was clozapine, which to this day is considered the “gold standard” in terms of efficacy [55]. However, the underlying mechanism for clozapine’s superior efficacy remains largely unknown and its clinical use is limited by the risk of developing agranulocytosis (observed in ~1% of patients) [56]. Notably, even though antipsychotics target positive symptoms, non-response of positive symptoms to antipsychotic treatment is common. For example, treatment-resistant schizophrenia (non-response to ≥2 medications) occurs in up to 34% of patients [57]. In addition, relapse of illness is common and occurs in approximately 30% of patients within a year of their first episode of psychosis, and in up to 80% of patients over the course of 5 years [58]. 

The urgency to develop new medications that treat the spectrum of schizophrenia symptoms with improved safety and tolerability is apparent [38,39]. In the last two decades, substantial investments have been directed towards the discovery and development of treatments with non-D_2_ receptor-based mechanisms of action, including candidate medicines with glutamatergic, serotonergic, cholinergic, neuropeptidergic, hormone-based, other dopaminergic (i.e., D_1_, D_3_), metabolic, histaminergic, infection/inflammation-based, and other mechanisms (reviewed in Girgis et al.) [59]. Despite several promising targets (e.g., allosteric modulation of NMDA receptor and ongoing work with muscarinic M_1_ and M_4_ receptor agonists), none of these mechanisms have yet yielded new medicines for schizophrenia patients. 

In recent years, substantial evidence has accrued, and two compounds have entered clinical development centered around a novel therapeutic target: trace amine-associated receptor 1 (TAAR1). In view of these new developments, we review TAAR1 pharmacology, candidate ligands in clinical development and the rationale for TAAR1 as a promising therapeutic target for neuropsychiatric disorders, focusing on schizophrenia.

## 2. TAAR1 As a Novel Therapeutic Target for the Treatment of Schizophrenia 

### 2.1. Trace Amines and TAAR1

**Figure 1:**
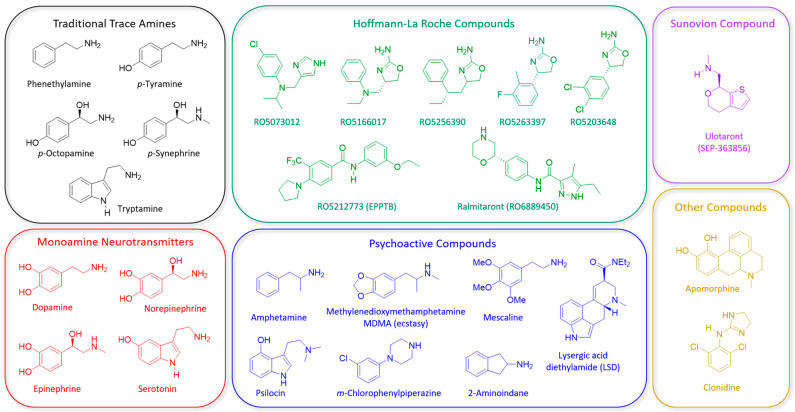
Structures of notable endogenous and synthetic TAAR1 ligands.

Trace amines (TAs) are a group of endogenous chemical messengers closely related to the biogenic amine neurotransmitters dopamine (DA), serotonin (5-HT) and norepinephrine (NE). TAs are found in both invertebrate and vertebrate species and are classically regarded as comprising β-phenylethylamine (β-PEA), p-tyramine, tryptamine, p-octopamine, and some of their metabolites (Figure 1) [60]. As suggested by their name, TA concentrations in the central nervous system are low, in fact, several hundred-fold lower than those of classical monoamine neurotransmitters [60,61]. 

Since the discovery of the first vertebrate trace amine-associated receptor, TAAR1, in 2001 [62,63], there has been an increasing interest in this G-protein coupled receptor as a novel target for the pharmacotherapy of various disorders including psychiatric illness [64,65,66]. This is largely attributed to TAAR1′s ability to modulate monoaminergic neurotransmission and behavior in rodents. In addition to the traditional TAs, TAAR1 is also activated by an array of other endogenous compounds including the monoamine neurotransmitters dopamine, serotonin, norepinephrine and some of their metabolites (Figure 1) [64,65].

The TAAR family consist of 6 functional members in humans, with TAAR1 being the most studied [67,68]. All human TAAR genes cluster to a small genomic region of 108 kb located in chromosome 6q23, which has been consistently identified as a susceptibility locus for schizophrenia and affective disorders [69]. Although several groups have attempted to identify susceptibility loci for mental disorders in TAARs, none of the reports received sufficient replication and the detected variants were not genome-wide significant. In contrast, several rare variants in TAAR genes (particularly TAAR1) have been detected in patients with mental and metabolic disorders (reviewed by Rutigliano and Zucchi) [66]. Some of the variants have demonstrated altered receptor function in vitro [69,70], warranting further assessment of naturally occurring TAAR1 variants in both brain and metabolic disorders. 

The regional and cell type-specific expression of TAAR1 is an on-going topic of debate given its generally low endogenous expression levels in the brain, and the lack of suitable, commercially available tools for labeling, such as specific antibodies [71]. Notwithstanding this, in rodents, TAAR1 mRNA and protein expression has been reported in monoaminergic nuclei of the brain including the ventral tegmental area (VTA), substantial nigra (SN), and dorsal raphe nucleus (DRN) [62,72]. Receptor expression has also been detected in limbic areas (e.g., amygdala, subiculum), basal ganglia, and the PFC. Thus, TAAR1 is ideally positioned to modulate dopaminergic, serotonergic and glutamatergic signaling and consequently regulate aspects of reward-processing, cognition and mood relevant to schizophrenia and other metal disorders. However, a comprehensive assessment of TAAR1 expression in the human brain is still lacking. Outside the CNS, TAAR1 expression in rodents and humans has been reported in pancreatic β-cells, the stomach, the intestines, and leukocytes [73,74,75,76,77,78,79,80,81,82,83], further suggesting the potential for TAAR1 as a target in metabolic and immune disorders. 

To date, the precise synaptic and cellular localization of TAAR1 is not clear, and additional studies are needed to elucidate both in cell systems and in vivo. Both TAAR1-mediated pre- and post-synaptic effects have been observed [84,85,86,87], which has complicated interpretation of some in vivo findings. Receptor localization has largely been reported intracellularly, with evidence for plasma membrane expression following ligand-induced heterodimerization with other GPCRs [63,67,74,76,88,89,90,91]. 

Although its interaction partners and downstream targets have not been fully elucidated, several groups have provided important insights into TAAR1-mediated signaling (reviewed in Berry et al., 2017; Gainetdinov et al., 2018.; Rutigliano et al., 2020) [64,65,66]. TAAR1 is a Gαs-coupled receptor that promotes cAMP production via stimulation of adenylyl cyclase [62,63,67], which can, in turn, promote protein kinase A (PKA) and protein kinase C (PKC) phosphorylation [92]. TAAR1 activation is also able to stimulate G protein-coupled inwardly rectifying potassium channels, which is proposed to underly TAAR1-mediated reduction in VTA dopaminergic neuron firing [84,86]. In addition, TAAR1 can signal via a G-protein independent, ẞ-arrestin2-mediated pathway [91], which appears most pronounced upon heterodimerization with D2 receptors. Differential signaling through physical interaction (i.e., heterodimerization) between TAAR1 and other GPCRs has been proposed. This includes interactions with 5-HT_1B_ [93], α_2A_ [94], potentially 5-HT_1A_ [86], and most prominently D_2_ receptors [87,90,91]. The interaction of TAAR1 and D_2_ receptors has been shown to exert functional effects both pre- and post-synaptically (reviewed in Gainetdinov et al., 2018; Berry et al., 2017; Rutigliano et al., 2020) [64,65,66]. Interestingly, heterodimerization of TAAR1 and D2 enhances the TAAR1-ẞ-arrestin2 interaction which ultimately results in reduced GSK3ẞ activation [91]. The proposed shift from cAMP accumulation to β-arrestin2 recruitment could have important pharmacological implications given that the AKT/GSK3β pathway is increasingly implicated in the pathophysiology of schizophrenia, bipolar disorder, and depression [65]. 

### 2.2. Development of Synthetic TAAR1 Ligands 

A challenge associated with the study of TAAR1 is its very broad ligand tuning [64,65]. As mentioned earlier, notable endogenous ligands of TAAR1 include the trace amines β-phenylethylamine (PEA), p-tyramine (TYR), and tryptamine as well as monoamine neurotransmitters dopamine, serotonin, and norepinephrine (Figure 1) [60,62,63,95]. In addition, various synthetic compounds are also reported to activate TAAR1 including psychoactive substances (e.g., amphetamine-like compounds, psilocin, etc.), apomorphine, and clonidine. Importantly, species differences with respect to potency and efficacy have been described for several of the mentioned synthetic ligands [63,96]. 

**Figure 2:**
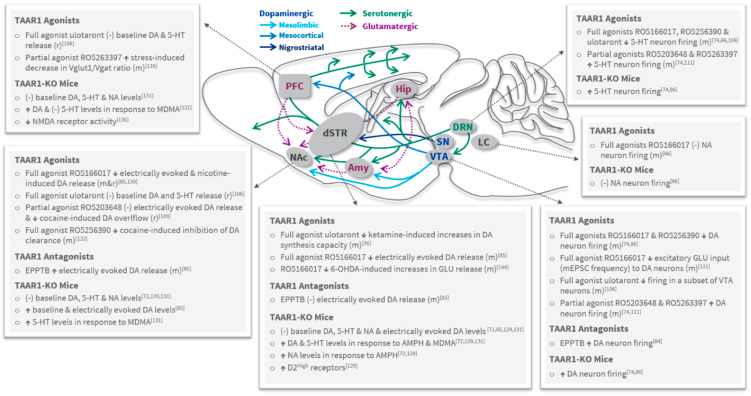
TAAR1-mediated modulation of monoaminergic and glutamatergic circuits. Simplified schematic of key serotonergic, dopaminergic, and glutamatergic pathways in the rodent brain including evidence from studies with synthetic ligands and TAAR1-KO mice. ↑, increase; ↓, decrease; (−), no change. Abbreviations: 5-HT (serotonin), AMPH (amphetamine), Amy (amygdala), dSTR (dorsal striatum/caudoputamen), DA (dopamine), Hip (hippocampus), LC (locus ceruleus), m (mouse), MDMA (3,4-Methylenedioxymethamphetamine), NA (noradrenalin), NAc (nucleus accumbens), PFC (prefrontal cortex), r (rat), SN (substantia nigra), VTA (ventral tegmental area).

Endogenous TAs exert effects at targets other than TAAR1, including aminergic and non-GPRC receptors as well as transporters [65,86,97]. Thus, the development of selective, small molecule TAAR1 ligands was crucial to advance our understanding of specific, TAAR1-mediated biological effects (Figure 1 and Table 2). This was initially explored by Hoffmann–La Roche, resulting in the development of highly selective, TAAR1 full and partial agonists based on different chemical scaffolds [98,99,100]. To date, five selective TAAR1 agonists have been extensively profiled preclinically (RO5166017, RO5073012, RO5256390, RO5203648 and RO5263397) (Table 2 and Figure 1 and Figure 2). These compounds have demonstrated antipsychotic, anti-addictive, pro-cognitive, antidepressant-like, and wake-promoting effects in rodents, likely via TAAR1-mediated modulation of dopaminergic, serotonergic and glutamatergic circuits [64,65,66]. A particular challenge has been the development of selective TAAR1 antagonists, and so far only one compound (EPPTB [-N-(3-ethoxyphenyl)-4-(1-pyrrolidinyl)-3-(trifluoromethyl)benzamide]) has been identified and characterized [84]. 

The Roche TAAR1 agonists have been studied as both basic research tools and as potential drug candidates. The partial agonist RO5263397 has progressed to Phase 1 clinical trials, however, identification of a population of poor metabolizers of RO52263397 has slowed further development of this compound [101]. Another partial agonist, ralmitaront (RO6889450), has completed first-in-human studies and is currently being evaluated for the treatment of schizophrenia in Phase 2 randomized, placebo-controlled clinical trials (ClinicalTrials.gov Identifiers NCT03669640 and NCT04512066) [102,103,104,105]. 

To date, the only TAAR1 agonist that has progressed to Phase 3 clinical trials is ulotaront (SEP-363856; Sunovion Pharmaceuticals), which was granted Breakthrough Therapy Designation from the U.S. Food and Drug Administration for the treatment of schizophrenia (Table 2 and Figure 1). Ulotaront was discovered through a unique, target-agnostic approach that was optimized to identify drug candidates that lack D_2_ and 5-HT_2A_ receptor antagonism while demonstrating an in vivo phenotypic antipsychotic-like profile [106]. Although the ulotaront’s mechanism of action has not been fully elucidated, subsequent in vitro and in vivo studies demonstrated that full agonism at TAAR1 and partial agonism at 5-HT_1A_ receptors are integral to its efficacy [106]. In addition, ulotaront shows partial agonism at 5-HT_1D_ and weak functional activity at 5-HT_7_ receptors. The fact that an independent, target-agnostic approach also identified TAAR1 agonism as an antipsychotic-like mechanism is further validation of TAAR1 as a promising therapeutic target for schizophrenia. 

**Table 2:** Pharmacological effects of TAAR1 agonists in preclinical species.

Compound (Company)	Human Receptor Profile	Behavioral Effects in Preclinical Models &Assays Relevant to Positive, Negative and Cognitive Symptoms of Schizophrenia	Clinical Trials in Schizophrenia Patients
RO5166017 F. Hoffmann-La Roche	TAAR1 Full Agonist [86]	↓ L-687,414-induced hyperactivity (mouse) [86] ↓ cocaine-induced hyperactivity (mouse) [86] ↓ hyperactivity in DAT-KO mice [86] ↓ cocaine-induced CPP (rat) [107] ↓ cue- and priming-induced reinstatement of cocaine-seeking (rat) [108] anxiolytic effect in SIH (mouse) [86]	N/A
RO5256390 F. Hoffmann-La Roche	TAAR1 Full Agonist [74]	↓ L-687,414-induced hyperactivity (mouse) [74] ↓ PCP-induced hyperactivity (mouse) [74] ↓ cocaine-induced hyperactivity (mouse and rat) [74] pro-cognitive effect in attentional set-shifting (rat) and object retrieval tasks (monkey) [74] antidepressant-like effect in differential reinforcement of low-rate behavior (monkey) [74] ↓ context-induced cocaine relapse (rat) [109] no antidepressant- or anxiolytic-like effects in FST & defensive withdrawal test (rat) [74,110] ↓ haloperidol-induced catalepsy (rat) [74]	N/A
RO5203648 F. Hoffmann-La Roche	TAAR1 Partial Agonist [111]	↓ L-687,414-induced hyperactivity (mouse) [111] ↓ cocaine-induced hyperactivity, self-administration & relapse (mouse/rat) [110,111] ↓ d-AMPH-induced hyperactivity in rats, no effect in mice [112] ↓ mAMPH-induced hyperactivity and self-administration (rat) [112] ↓ hyperactivity in DAT-KO mice and rats [109,113] ↓ hyperactivity in NR1-KD mice [111] pro-cognitive effect in object retrieval tasks (monkey) [111] antidepressant-like effect in FST (rat) and differential reinforcement of low-rate behavior (monkey) [111] modest anxiolytic effect in SIH (mouse) [111] ↓ haloperidol-induced catalepsy (rat) [111]	N/A
RO5263397 F. Hoffmann-La Roche	TAAR1 Partial Agonist [74]	↓ L-687,414-induced hyperactivity (mouse) [74] ↓ PCP-induced hyperactivity (mouse) [74] ↓ cocaine-induced hyperactivity (mouse) [74] ↓ abuse-related effects of cocaine, mAMPH, nicotine and morphine (rat) [107,114,115,116,117] ↓ cocaine-induced ICSS (rat) [118] reversed chronic stress-induced cognitive and social interactions deficits without producing effects on anxiety in the EPM (mice) [119] ↑ mismatch negativity (rat) [120] pro-cognitive effect the object retrieval task (monkey) [74] antidepressant-like effect in FST (rat) and differential reinforcement of low-rate behavior (monkey) [74] ↓ haloperidol-induced catalepsy (rat) [74]	N/A
RO5073012 F. Hoffmann-La Roche	TAAR1 Partial Agonist [121]	↓ AMPH-induced hyperactivity in *Taar1* overexpressing mice–no effect in WT mice [121]	N/A
Ralmitaront (RO6889450) F. Hoffmann-La Roche	TAAR1 Partial Agonist	N/A	Two Phase 2 studies ongoing DB, pbo-controlled in patients with an acute exacerbation of schizophrenia or schizoaffective disorder (NCT04512066) [102] DB, pbo-controlled in patients with negative symptoms of schizophrenia (NCT03669640) [103]
Ulotaront (SEP-363856) Sunovion Pharmaceuticals	TAAR1 Full Agonist and 5-HT_1A_ Partial Agonist [106]	antipsychotic-like behavioral profile in the SmartCube platform (mouse) [106] ↓ PCP-induced hyperactivity (mouse/rat) [106,122] no effect on AMPH-induced hyperactivity (rat) [122] ↓ PCP-induced social interaction deficits (rat) [106] ↑ prepulse inhibition (mouse) [106] improved object recognition memory in subchronic PCP-treated rats [122] ↓ ketamine-induced increase in striatal dopamine synthesis capacity (mouse) [36] modest antidepressant effects in the mouse FST [106]	Two Phase 2 studies completed: DB, pbo-controlled in patients with an acute exacerbation of schizophrenia demonstrated efficacy and good tolerability [123] 26-week open-label extension study demonstrated continued effectiveness and long-term tolerability [124] Four Phase 3 studies ongoing: Two DB, pbo-controlled studies in patients with an acute exacerbation of schizophrenia [125,126] 52-week open-label extension study [127] 52-week long-term safety study [128]

**Abbreviations:** (**↑**) increase and (↓) decrease relative to vehicle control. Amphetamine (AMPH), double-blind (DB), conditioned place-preference (CPP), dopamine transporter knockout (DAK-KO), elevated plus-maze (EPM), forced swim test (FST), placebo (pbo), intracranial self-stimulation (ICSS), methamphetamine (mAMPH), not available (N/A), phencyclidine (PCP), stress-induced hyperthermia (SIH), subchronic (sc), wild-type (WT).

## 3. Preclinical Evidence for TAAR1 as a Therapeutic Target for Schizophrenia

In this section, we summarize preclinical evidence supporting TAAR1 agonists as a potential therapeutic candidate in schizophrenia that may offer differentiation from antipsychotics, which are limited to the efficacy and associated safety profile of D_2_ antagonism. We provide an overview of TAAR1′s role in regulating monoaminergic and glutamatergic circuits implicated in schizophrenia pathophysiology, and discuss the potential for TAAR1 agonists in psychosis, negative symptomatology, cognition, mood, and anxiety as they relate to schizophrenia. 

### 3.1. Role of TAAR1 in Psychosis and Dopaminergic Tone

Since the first studies in TAAR1-knockout (KO) mice [72,129] and initial findings that selective TAAR1 agonism exerts inhibitory effects on dopaminergic and serotonergic neuronal activity [86], there has been a growing body of research characterizing the role of TAAR1 in the modulation of monoaminergic circuits (Figure 2). Particularly the ability of TAAR1 to regulate dopaminergic tone has been of interest in the context of schizophrenia and psychosis in general. In brain slices, selective, full TAAR1 agonists suppress VTA neuronal firing [74,86] and inhibit electrically evoked dopamine release in the nucleus accumbens (NAc) [85]. Similarly, ulotaront (TAAR1 agonist with 5-HT_1A_ agonist activity) was found to decrease VTA neuronal firing, although this was only observed in a subset of neurons [106]. Consistent with this, the firing rate of dopaminergic VTA neurons is increased in TAAR1-KO mice and enhanced by the TAAR1 antagonist EPPTB [72,84]. Interestingly, the partial agonists RO5203648 and RO5263397 also increase VTA DA neuron firing suggesting that, in vitro, TAAR1 is constitutively active and/or tonically activated by endogenous agonists, a situation where partial agonism produces a net antagonistic effect [74,111]. The extent to which such differential effects occur in vivo remains to be determined. While the molecular mechanism has not been fully elucidated, the effects on DA neuron firing may result from TAAR1 activation of inwardly rectifying K+ channels [84,86]. In addition, the full agonist RO5166017 was shown to decrease miniature excitatory post synaptic current (mEPSC) frequency in the VTA without altering the amplitude [121]. This suggests that TAAR1 is present in the pre-synaptic compartment of excitatory inputs to DA neurons and participates in agonist-mediated reduction of VTA DA neuron firing. 

Although TAAR1 ligands exert prominent effects on DA neuron firing in brain slices, less clarity exists on how this ultimately affects DA release in the striatum and other brain regions (Figure 2). Fast scan cyclic voltammetry (FSCV) experiments with selective agonists have demonstrated attenuation of electrically evoked DA release in brain slices in the NAc and dorsal striatum of mice [85], and in vivo in the NAc of rats [130]. On the other hand, in vivo microdialysis studies revealed no effects on baseline striatal DA release with ulotaront and inconsistent observations are reported in TAAR1-KO mice [72,85,106,131]. Prominent changes in monoaminergic neurotransmission were also observed in TAAR1 overexpressing mice although these effects are difficult to interpret due to the largely ectopic expression of the receptor [121]. In addition, Leo et al., showed that lack of TAAR1 leads to increased DA release predominately in the NAc, suggesting that TAAR1 may exert differential regulatory effects in the NAc compared to the dorsal striatum [85,121]. Further studies, particularly in the substantia nigra and PFC are required to decipher potential differences in TAAR1-mediated modulation of mesolimbic, mesocortical, and nigrostriatal dopaminergic circuits. The mechanism of TAAR1-dependent regulation of DA release is not fully understood but may involve physical interaction with presynaptic D_2_ receptors resulting in the modulation of autoinhibition [85,89,90,132]. 

While TAAR1 partial and full agonists can differentially modulate baseline DA neuron firing, consistent antipsychotic-like effects of both partial and full agonists have been reported in several rodent models of schizophrenia (Table 2). TAAR1 agonists robustly block hyperactivity induced by cocaine, amphetamine, PCP and L-687,414 in rodents [74,86,106,111,112,122], likely through direct effects on DA neurotransmission and/or upstream modulation of glutamatergic activity. Thus, various TAAR1 agonist profiles are effective in paradigms based on hyperdopaminergic activity (i.e., cocaine, amphetamine) and NMDA receptor hypofunction (PCP, L-687,414), similar to antipsychotics. Importantly, the antipsychotic-like efficacy of selective agonists was absent in TAAR1-KO mice, confirming the contribution of the receptor to the behavioral effects [74,86]. On the other hand, an exaggerated response to the dopaminergic stimulant amphetamine was observed in TAAR1-KO mice, both in terms of hyperlocomotor activity and striatal dopamine release [72,129], indicative of a schizophrenia-like dopamine phenotype. Furthermore, TAAR1 agonists inhibit dopamine-dependent hyperactivity in DAT-KO mice and rats [86,111,113,121] and prevent cocaine-induced dopamine overflow in the NAc [109]. In addition, the TAAR1 full agonist RO5256390 blocks cocaine-induced inhibition of DA clearance in slices of the NAc. This was dependent on simultaneous D2 autoreceptor activation and associated downstream GSK-3ẞ signaling, likely facilitated through TAAR1-D_2_ heterodimerization [132]. In light of the current data, TAAR1-mediated suppression of DA neurotransmission appears to be most prominent under hyperdopaminergic conditions. This is further supported by recent results showing that ulotaront attenuates the ketamine-induced increase in striatal dopamine synthesis capacity without producing an effect in naïve mice [36]. This is relevant considering that elevated dopamine synthesis capacity has been reported in schizophrenia patients and is not targeted by current antipsychotic treatments [25,27,133,134]. Whether ulotaront’s effects on DA synthesis capacity are mediated through direct action on dopaminergic neurons, or occur further upstream, remains to be elucidated. Notwithstanding this, the preclinical evidence to date suggests that TAAR1 agonism may represent a unique approach to modulate the presynaptic DA dysfunction observed in psychosis. 

### 3.2. Role of TAAR1 in Cognition, Negative Symptoms, Mood and Anxiety 

As described above, cognitive deficits, negative symptoms, and affective symptoms of schizophrenia are poorly treated by current antipsychotic medications. While the pathophysiology underlying these symptoms is not fully understood, hypofunctioning of the PFC is thought to contribute to them [135]. Several preclinical studies have investigated the role of TAAR1 in PFC functioning as well as the effect of TAAR1 modulation on cognition, mood- and anxiety-related behaviors (Table 2 and Figure 2). In non-human primates (NHPs), TAAR1 agonists have been reported to increase accuracy in the object retrieval task indicative of improved cognitive performance [74,111]. Further pro-cognitive effects were shown for RO5256390, which reversed PCP-induced deficits in executive function in the rat attention set shift task [74]. In addition, RO5263397 improved chronic stress-induced deficits in cognitive function as well as the associated changes in dendritic arborization and synapse number in the PFC [119]. Interestingly, specific deletion of TAAR1 in the mPFC mimicked some of the cognitive deficits induced by chronic stress [119]. In contrast, no pronounced alterations in cognitive performance were reported for TAAR1-KO mice, although aberrant perseverative and impulsive behaviors were observed [129,136]. The discrepancy is likely caused by differences in temporal and spatial specificity of receptor deletion (selective receptor ablation during adulthood vs. global deletion embryonic development). Thus, compensatory developmental effects cannot be excluded in TAAR1-KO mice. Additional support for antipsychotic and pro-cognitive effects of TAAR1 agonists comes from studies assessing prepulse inhibition (PPI) and mismatch negativity (MMN). Impairments in PPI and MMN have repeatedly been observed in schizophrenia patients and are considered a potential biomarker of the disease [137,138,139,140,141,142]. Particularly deficits in MMN are believed to reflect cognitive decline and have been associated with glutamatergic NMDA receptor alterations [139,142]. In naïve mice, ulotaront and RO5263397 increase PPI and MMN, respectively [120]. Whether TAAR1 agonists can reverse impairments in PPI and MMN (e.g., in rodent models of schizophrenia) remains to be investigated. 

In contrast to positive and cognitive symptoms, negative and affective symptoms of schizophrenia are more difficult to model in animals. Thus far, only a few studies have investigated the role of TAAR1 in social, anxiety and depression-related behavior. Ulotaront reversed PCP-induced social interaction deficits in rats, suggesting potential benefits against some of the negative symptoms of schizophrenia, such as social withdrawal [106]. TAAR1 agonists have also been assessed for antidepressant-like activity in the classical forced swim test (FST). While partial agonists RO5263397 and RO5203648 decreased immobility in the rat FST, no effect was observed with the full TAAR1 agonist RO5256390 [74,110,111]. In contrast, modest activity was reported for the full TAAR1 agonist ulotaront in the mouse FST, although a contribution of 5-HT_1A_ agonism to this effect cannot be excluded [106]. Positive effects of TAAR1 agonists were also observed on Differential Reinforcement of Low-Rate Behavior (DRL) in NHPs, an antidepressant-sensitive paradigm that engages PFC and hippocampus activity [74,111]. In addition, TAAR1 activation in mice attenuates stress-induced hyperthermia [86,111] and improves chronic stress-induced social avoidance [119], suggesting potential antidepressant and/or anti-anxiety effects. In contrast, no effects of TAAR1 activation on anxiety-related behavior were reported in the defensive withdrawal test in naïve rats [110] or in the elevated plus-maze in chronically stressed mice [119]. 

The above studies encourage further assessment of pro-cognitive, and putative anxiolytic and antidepressant-like properties of TAAR1 agonists, not only in the context of negative symptoms of schizophrenia, but also other psychiatric disorders. 

### 3.3. TAAR1 Effects on Serotonergic, Noradrenergic and Glutamatergic Systems 

Effects of TAAR1 agonists on serotonergic and glutamatergic systems have been reported (Figure 2), although the underlying molecular mechanisms have not been explored in detail. Similar to the findings in the VTA, full and partial agonists increase and decrease serotonergic neuronal firing in the DRN, respectively [74,86]. These effects were TAAR1-dependent as they were not observed in TAAR1-KO mice and reversed by the antagonist EPPTB [74,86]. Although ulotaront was also reported to decrease firing in the DRN in vitro and in vivo, these effects were largely attributed to 5-HT_1A_ receptor activation [106]. Interestingly, ulotaront did not alter baseline extracellular serotonin levels in the striatum and PFC of rats [106]. Whether selective TAAR1 agonists modulate serotonin release in vivo remains to be determined. Compared to wild-type controls, TAAR1-KO mice exhibit increased spontaneous firing of DRN neurons and enhanced striatal serotonin release in response to an amphetamine challenge [72,74,86]. However, no significant changes in baseline serotonin release were detected in the dorsal striatum, NAc or PFC of TAAR1-KO mice [131]. Notably, Revel and colleagues reported that TAAR1 activation increases agonist potency at 5-HT_1A_ receptors in brain slices of the DRN. In addition, TAAR1 is required for the desensitization of 5-HT_1A_ autoreceptors [86]. Given that desensitization of the 5-HT_1A_ autoreceptor has been linked to the efficacy of antidepressants, co-treatment with TAAR1 agonists might further improve the therapeutic effects of these agents. Although additional studies are necessary to further elucidate the potential interaction between TAAR1 and 5-HT_1A_ in vitro and in vivo, compounds with dual TAAR1/5-HT_1A_ activity may provide additional benefits in the treatment of psychosis, mood and anxiety. 

Currently, there is little evidence for TAAR1-mediated modulation of the noradrenergic system. In contrast to its effect in the VTA and DRN, the full agonist RO5166017 did not alter the firing frequency of noradrenergic neurons of the locus coeruleus [86]. This is in agreement with the reported absence of detectable TAAR1 expression in the locus coeruleus [72], although a more recent study suggests that TAAR1 may regulate glutamate signaling in cultured noradrenergic neurons [143]. Studies in TAAR1-KO mice reported no changes in noradrenergic neuron firing or baseline noradrenaline levels in the PFC, dorsal striatum and NAc compared to wild type controls [86,131]. However, TAAR1-KO mice exhibit enhanced striatal noradrenaline release in response to an amphetamine challenge [72]. Thus, in summary, evidence is mixed, and additional studies are needed to further elucidate whether TAAR1 can directly regulate noradrenergic activity. 

As previously described, TAAR1 agonists can attenuate the behavioral deficits induced by NMDA receptor antagonists PCP, L-687,414 and ketamine [74,86,106,111]. The molecular basis of this is not well understood and may involve direct modulation of glutamatergic neurotransmission, or downstream effects on dopaminergic circuits. TAAR1-mediated modulation of cortical glutamatergic transmission has been of particular interest as its deficiency is implicated in the pathophysiology of schizophrenia [30]. As mentioned, TAAR1 agonism can attenuate chronic stress-induced cognitive impairments possibly by restoring excitatory/inhibitory imbalance and structural alterations in the mPFC [119]. Along these lines, TAAR1-KO mice exhibit reduced NMDA receptor activity in the mPFC layer V neurons, as suggested by alterations in evoked EPSC kinetics and receptor subunit composition, as well as reduced mEPSC amplitude and frequency [136]. The observed glutamate transmission deficits could be a direct consequence of the absence of cortical TAAR1 and/or may be secondary to alterations in the cortical dopamine system [136]. The ability to impact the interaction between glutamatergic and dopaminergic circuits may underlie the beneficial behavioral effects of TAAR1 agonists in rodent models of psychiatric disorders. This is supported by slice electrophysiology experiments, suggesting that TAAR1 can decrease VTA neuron firing not only through direct action on DA neurons, but also by reducing glutamatergic, excitatory inputs to DA neurons [121]. Intriguingly, TAAR1 has also been shown to impact pre- and post-synaptic glutamatergic neurotransmission in the striatum of rodent Parkinson’s disease models [144]. The ability to regulate corticostriatal crosstalk is not only of relevance to Parkinson’s disease, but also schizophrenia and other psychiatric disorders [145]. Moreover, TAAR1 may participate in glutamate clearance via modulation of the excitatory amino acid transporter 2 (EAAT2) [146,147].

## 4. Additional Considerations for TAAR1 Agonists as Therapeutic Agents for Schizophrenia 

In contrast to antipsychotics, and consistent with the lack of D2 receptor activity, neither RO5256390, RO5263397 nor ulotaront induced catalepsy in rodents [74,106]. On the contrary, RO5263397 even partially prevented haloperidol-induced catalepsy, suggesting that TAAR1 partial activation may mitigate EPS caused by neuroleptics [74]. Interestingly, the same authors also showed that TAAR1 agonism can potentiate the antipsychotic properties of olanzapine and risperidone, highlighting the potential of TAAR1 agonists as adjunctive treatments to current antipsychotics [74]. Thus, although TAAR1 agonists demonstrate antipsychotic-like efficacy in schizophrenia models, they are unlikely to cause extrapyramidal side effects (EPS, movement disorders), which are a well-known side effect of current antipsychotics. This is also supported by recent clinical results with ulotaront (discussed in detail below). While encouraging, further validation of these findings is required. 

Importantly, a growing body of evidence implicates TAAR1 in the regulation of metabolic function and food reward behavior, which has been extensively reviewed elsewhere [65,66,148]. This is of significant relevance considering that weight gain and altered levels of several metabolic parameters constitute major side effects of antipsychotic medication [42]. As noted, antipsychotic-induced weight gain can increase the risk of cardiovascular side effects and diabetes and result in treatment discontinuation and overall reduced quality of life [149,150]. In contrast to current antipsychotics, TAAR1 agonists do not increase body weight in naïve rodents, but rather tend to decrease it [74,76]. In addition, TAAR1 agonists were shown to prevent olanzapine-induced weight gain in rats [74] and reduce food intake and excess body weight in diet-induced obese mice [76]. Further studies in rodent models of type 2 diabetes mellitus revealed improved glucose tolerance and insulin sensitivity, as well as reduced plasma and liver triglyceride levels [76]. The observed metabolic effects are likely mediated by peripheral TAAR1 activation in pancreatic ẞ-cells and enteroendocrine cells of the intestine. On the other hand, TAAR1-mediated regulation of food intake and food-reward behavior has been linked to its central effects, predominately dopaminergic and/or glutamatergic in origin [148,151]. This is supported by the initial work of Ferragud and colleagues demonstrating that the TAAR1 agonist RO5256390 (administered systemically or microinfused in the mPFC) prevents binge-like eating of palatable food in rats [110]. 

Taken together, the current preclinical data suggest that TAAR1 agonists have the potential to improve several symptom domains of schizophrenia without causing adverse effects such as motor impairments or weight gain. Furthermore, the beneficial metabolic and antidiabetic effects of TAAR1 agonists raise the question of whether these agents may be uniquely positioned to improve comorbid metabolic disorders in patients with schizophrenia.

## 5. Clinical Evidence for TAAR1 Agonists for the Treatment of Schizophrenia

Two TAAR1 agonists have advanced to clinical trials in patients with schizophrenia: ulotaront and ralmitaront (Table 2; Figure 1). Pharmacokinetic (PK) studies in humans have been completed with both agents, but results are available only for ulotaront. In population PK analyses at dose levels ranging from 10–100 mg, ulotaront is well-absorbed and exhibits linear PK dose-proportionality with a median Tmax of 2.8 h and an effective half-life of 7 h [152]. 

Ralmitaront is currently in Phase 2 development with two double-blind, placebo-controlled clinical studies examining the efficacy and safety of ralmitaront in patients with schizophrenia. One study is designed to evaluate two doses of ralmitaront in patients aged 18 to 45 with an acute exacerbation of schizophrenia or schizoaffective disorder over 4 and 12 weeks (NCT04512066), while the other aims to assess one dose of ralmitaront over 12 weeks in patients aged 18 to 55 with negative symptoms of schizophrenia as both a monotherapy and as adjunctive therapy to D_2_ antagonists or D_2_/5HT_2A_ dual antagonists (NCT03669640) [102,103]. 

Ulotaront has progressed to Phase 3 development after favorable efficacy and tolerability data from two completed Phase 2 trials. The ongoing Phase 3 studies include two double-blind, placebo-controlled studies, one enrolling patients aged 12–65 and the other 18–65, which each aim to assess the efficacy and safety of fixed doses of ulotaront in patients with an acute exacerbation of schizophrenia (NCT04072354 and NCT04092686) [125,126], a 52-week, open-label, flexible-dose extension study, (NCT04109950) [127] and another 52-week study evaluating ulotaront compared to quetiapine XR in patients with stable schizophrenia (NCT04115319) [128]. 

The completed Phase 2 studies included a 4-week, randomized, double-blind, placebo-controlled study evaluating efficacy and safety of flexibly dosed ulotaront (50 to 75 mg/day) in patients aged 18–40 with an acute exacerbation of schizophrenia [123], and a 26-week, open-label extension study in patients completing the 4-week double-blind study [124]. The dose selection for Phase 2 clinical trials was guided by an initial study in healthy male volunteers demonstrating robust REM sleep-suppressing effects of ulotaront at 50 mg [153].

In the 4-week, double-blind, placebo-controlled study, treatment with ulotaront resulted in a significantly greater reduction from baseline in the Positive and Negative Syndrome Scale (PANSS) total score for ulotaront than placebo at Week 4 (Least-Squares Mean change from Baseline to Week 4 [SE]: −17.2 [1.7] vs. −9.7 [1.6]; *p* = 0.001; effect size [ES] = 0.45). Robust improvements relative to placebo were also observed on the PANSS positive, negative, and general psychopathology subscale scores as well as other secondary endpoints including the Clinical Global Impression of Severity (CGI-S) score, the Montgomery-Asberg Depression Rating Scale (MADRS) total score and the Brief Negative Syndrome Scale (BNSS) total score, a scale designed to specifically assess the negative symptoms of schizophrenia. Effects on several of the BNSS subscale scores at Week 4 were also observed, including on the Alogia, Asociality, Anhedonia, Avolition and Blunted Affect subscales and on the negative symptom factors of the Uncorrelated PANSS Score Matrix transformation of the PANSS (UPSM-PANSS) [154,155]. The UPSM-PANSS factors have been shown to have low levels of between-factor correlation across multiple clinical trials in schizophrenia [156]. This suggests that these UPSM factors are detecting a true negative symptom treatment effect for ulotaront and not a nonspecific effect secondary to improvement in correlated PANSS items. Thus, these findings suggest potential benefits of TAAR1 agonists for negative symptoms. 

In the 26-week open-label extension study [124] where all patients received flexibly dosed ulotaront (25 to 75 mg/day), continued improvement across a broad array of schizophrenia symptoms was observed, as measured by the PANSS total score and subscale scores (positive, negative and general psychopathology), CGI-S score, MADRS total score and BNSS total score. Furthermore, treatment response rates at Week 26 (as measured by a ≥30% reduction in PANSS total score from the baseline of the double-blind study) were high (94.1% for patients who received ulotaront in the double-blind study and continued ulotaront treatment in the extension study, and 92.5% for patients who initially received placebo in the double-blind study and were switched to ulotaront in the extension study [observed cases]). Of the 156 patients who received ulotaront in the extension study, 67% completed 26 weeks of treatment, a rate notably higher than 37–49% attrition rate reported previously in 6-month extension studies of many current antipsychotics [54,157]. 

The completed Phase 2 studies with ulotaront suggest that it is effective in treating the symptoms of schizophrenia with a clinical safety and tolerability profile that lacks the class-related side effects of currently marketed antipsychotics. In the 4-week, double-blind study, the safety and tolerability of ulotaront was shown to be similar to the placebo. The incidence of adverse events (AEs) was generally similar in the ulotaront and placebo groups, with the only AEs reported in ≥2% of the ulotaront group and twice the rate of the placebo group being diarrhea (2.5% in ulotaront and 0.8% in placebo) and dyspepsia (2.5% in ulotaront and 0 in placebo). Notably, treatment with ulotaront was not associated with EPS or other movement disorders, supporting its lack of D_2_ blockade. Percentages of patients reporting EPS AEs (3.3% ulotaront; 3.2% placebo) and results on scales used to measure motor impairments (AIMS, BARS, SAS) were similar between groups. Mean changes in metabolic laboratory parameters and prolactin were also similar to placebo and there were no clinically significant ECG findings. The results of the 26-week extension study were consistent with these results. Overall, there was a low incidence of AEs. AEs related to EPS were few and there were no clinically meaningful changes on scales used to measure the degree of motor symptoms. In addition, ulotaront had no clinically meaningful effects on weight, lipids, glycemic indices, prolactin, or ECG parameters (including no evidence for prolongation of the QTc interval)—all hallmark side effects associated with antipsychotics that work via D_2_ receptor antagonism. Taken together, these results suggest a generally well-tolerated safety profile for ulotaront that is consistent with the absence of D_2_-receptor blockade and supportive of a novel mechanism of action and a new drug class for the treatment of schizophrenia. 

## 6. Conclusions

Foundational preclinical research and recent clinical evidence demonstrate the promise of TAAR1 agonists to be the first novel drug class for the treatment of schizophrenia in almost 70 years. Ulotaront recently reached recommended status for its proposed International Nonproprietary Name (INN), joining TAAR1 partial agonist ralmitaront in this class with the designated stem “-taront” [158]. These recent developments have ignited further interest in TAAR1 biology. Preclinical data suggest TAAR1 agonists may be uniquely positioned to target the presynaptic mechanisms underlying dopamine synthesis capacity dysfunction in psychosis and modulate glutamatergic circuit alterations associated with core symptoms of schizophrenia. Thus, increasing our knowledge of TAAR1 and its ability to modulate monoaminergic and glutamatergic circuits is not only important for the development of future TAAR1 agonists and their potential therapeutic effects, but possibly also to our understanding of the pathophysiology of schizophrenia. Future work should be aimed at pinpointing the precise cellular and subcellular localization of TAAR1 and increasing our understanding of its signal transductions and molecular interactions. 

The potential for a non-D_2_-based approach to offset the last 70 years of treatment with antipsychotics is promising and underscores the need for treatments with broad-spectrum efficacy and improved safety and tolerability compared to current antipsychotics. To date, treatments indicated for schizophrenia have been restricted to drugs that primarily act via antagonism at D_2_ and/or 5-HT_2A_ receptors. Ongoing Phase 2 and 3 clinical trials will determine whether TAAR1 agonists can circumvent the fate of prior, non-D_2_ receptor experimental drugs to become the first novel mechanism for the treatment of schizophrenia. Although initial clinical results with ulotaront are encouraging, these findings will need to be replicated in additional clinical studies. 

Most currently available antipsychotics are associated with significant side effects, with EPS and cardiometabolic disturbances considered to be the most troublesome. Preclinical and clinical data to date suggest that TAAR1 agonists may improve several symptom domains of schizophrenia without causing debilitating motor impairments or metabolic syndrome. The availability of a treatment with a novel safety profile therefore becomes an attractive option. 

In general, the reduction of positive symptoms serves as a marker of currently defined treatment success with antipsychotics; however, other important symptoms of schizophrenia and comorbidities are often ignored or undertreated. Both preclinical and clinical studies and analyses support the hypothesis that TAAR1 agonists may be effective in treating the negative symptoms of schizophrenia. Moreover, studies in rodents and non-human primates have demonstrated pro-cognitive effects of TAAR1 agonists. The potential availability of an agent able to address negative and cognitive symptoms (notably present in the prodromal period) may enable earlier intervention especially if a differentiated risk-benefit profile vs. antipsychotics can be demonstrated. This notion however needs to be thoroughly explored, as these scenarios have not been investigated in clinical trials. Future studies examining the consequences of earlier intervention with TAAR1 agonists would be of interest.

TAAR1 may be a valuable therapeutic target in areas beyond schizophrenia and psychosis. A considerable body of preclinical evidence supports TAAR1 as a promising target for the treatment of metabolic syndrome and obesity, substance abuse, sleep disorders characterized by changes in REM sleep, and potentially mood and anxiety disorders. Considering that many of these represent comorbidities of schizophrenia, future studies should assess whether TAAR1 agonists have the potential to treat a spectrum of largely unaddressed schizophrenia symptoms which would otherwise require combination treatments and polypharmacy.

In the foreseeable future, findings from ongoing clinical trials with ralmitaront and ulotaront will be fundamental to elucidate the therapeutic utility of TAAR1 agonists, which hold great promise as a new drug class for the treatment of schizophrenia.

## Figures and Tables

**Table 1:** Schizophrenia is characterized by symptoms that can be grouped into positive, negative, and cognitive domains [2,3].

Positive Symptoms	Negative Symptoms	Cognitive Symptoms-Impairments in:
Delusions - Fixed, false belief	Blunted affect, diminished facial/vocal expression	Speed of processing
Hallucinations - Auditory - Visual - Tactile - Olfactory	Avolition, emotional withdrawal	Attention/vigilance
Disorganized thinking Disorganized behavior	Asociality, social isolation	Working memory
	Anhedonia, difficulty anticipating pleasurable activities	Verbal and visual learning
	Alogia, few words and avoidance of communication	Reasoning and problem solving
		Social cognition
